# Research on Urban Spatial Connection and Network Structure of Urban Agglomeration in Yangtze River Delta—Based on the Perspective of Information Flow

**DOI:** 10.3390/ijerph181910288

**Published:** 2021-09-29

**Authors:** Qiaowen Lin, Mengyu Xiang, Lu Zhang, Jinjiang Yao, Chao Wei, Sheng Ye, Hongmei Shao

**Affiliations:** 1 Department of Economics, School of Economics and Management, China University of Geosciences, Wuhan 430079, China; linqiaowen@cug.edu.cn (Q.L.); shaohm@cug.edu.cn (H.S.); 2 Chinese Research Academy of Environmental Sciences, Beijing 100012, China; xiangmy@cug.edu.cn; 3 School of Public Administration, Central China Normal University, Wuhan 430079, China; 4 Science and Technology Innovation and Public Management Research Center of Shanghai, Fudan University, Shanghai 200433, China; 5 Department of Land Administration, School of Public Administration, Hubei University, Wuhan 430062, China; weichao@hubu.edu.cn; 6 Department of Land Administration, School of Public Administration, China University of Geosciences, Wuhan 430079, China; yesheng@cug.edu.cn

**Keywords:** information flow, Yangtze River Delta urban agglomeration, spatial connection, the network structure

## Abstract

Exploration of urban spatial connections and network structures of urban agglomeration in the Yangtze River Delta, as well as its influencing factors, is of great significance regarding optimization of the development pattern of the Yangtze River Delta urban agglomeration and promotion of regional high-quality development. Therefore, based on Baidu index data in 2015 and 2019, this paper first analyzes the spatiotemporal variation characteristics of information-flow connections in the Yangtze River Delta urban agglomeration. Then it uses social network analysis to explore the information-flow network structure in the Yangtze River Delta urban agglomeration, and finally explores the influencing factors of information-flow intensity in the Yangtze River Delta urban agglomeration. The main conclusions are as follows: (1) The total amount of information flow in the Yangtze River Delta urban agglomeration has had no obvious change, and the coverage of information flow in the central urban circle has expanded. (2) The network hierarchy presents a relatively stable “pyramid” distribution pattern, which tends to develop into a “spindle” pattern. (3) The overall network density of the Yangtze River Delta urban agglomeration is high and is increasing. The backbone network is a “triangle” structure. The central cities in the region are stable, and the subgroups are adjacent to each other geographically. (4) Gross Domestic Product, resident population of the region and the number of Internet broadband subscribers all have important effects on the total information flow, among which the number of Internet broadband subscribers has the greatest effect on the total information flow. In addition, urban functions and their positioning, urban events, history and culture, and other factors that are difficult to quantify also have a certain impact on the information-flow network among cities.

## 1. Introduction

With the accelerated development of transportation and information, globalization and informatization have become the trend of social development. Geographical conditions have gradually reduced restrictions on regional exchanges. Capital and technology flows have been accelerated and countries are interconnected and interdependent in terms of economy, politics and trade, forming an organic whole on a global scale [1]. Under this general trend, the urban network across the boundaries of cities has gradually emerged, and derives large-scale cities, such as global cities and world cities. Based on this, it is difficult for any city to exist independently in the world urban system [2]. At the same time, the research focus of urban geography is shifted to regional spatial characteristics and urban network structure, covering both vertical and horizontal connections among cities [3,4]. 

The concept of “flow space” was first proposed in 1989 by Castells, an American scholar, in his book *Information City: Information Technology, Economy in Structure and Urban-regional Process*. Castells points out that more and more elements are juxtaposed across distances in real time in the global information network, and the “flow space” can be divided into four levels [5]. In urban geography, “flow space” refers to the establishment of new spatial logic based on the continuous movement of logistics, people flow, information flow, traffic flow, technology flow, capital flow and the main support of basic transportation facilities under the background of the information era. The high-speed characteristics of various “flow” elements make the spatial circulation of various elements more convenient and efficient, which greatly shortens the distance among cities and promotes the development process of urban space network [6]. With the advent of the era of big data, “flow” data can be measured more truly, making urban network exploration through “flow” elements more dynamic, scientific and intuitive [7,8]. 

As an important intersection of the Belt and Road Initiative and the Yangtze River Economic Belt, the urban agglomeration in the Yangtze River Delta plays an important strategic role in China’s overall modernization drive and opening-up pattern. It is an important platform for China to participate in international competition and an important engine for economic and social development, playing a leading role in the Yangtze River Economic Belt [9,10]. In this context, this paper uses the Baidu Index to explore the intercity connection of Yangtze River Delta urban agglomeration from the perspective of information flow. Social network analysis is used to analyze the urban network structure. The influencing factors of the spatial structure of Yangtze River Delta urban agglomeration are analyzed in order to understand deeper the urban connection and spatial structure of it, which provides useful guidance for the construction of regional planning. 

The rest of this article is organized as follows. The second section reviews the relevant literature. The third section introduces the research methods and data sources, which is followed by the empirical analysis. Last but not the least, conclusions and suggestions are given.

## 2. Literature Review

Urban systems and urban spatial distribution have always been the focus of geographical and urban planning. Early studies on urban networks mostly used data on urban statistical attributes, such as population, gross regional product and other single or multiple indicators to classify cities, which was relatively static research [11]. Based on static data, Pan et al. analyzed the spatial pattern and evolution of container liner network in the Yangtze River Delta [12]. Wu et al. explored the cyberspace characteristics of Chinese electronic information enterprises [13]. These studies enrich traditional identification of spatial patterns. 

However, since the 1980s, with the development of globalization, information and networking, urban flow space has affected the development of future urban spatial patterns to a certain extent [14]. Castells was the first to propose that society is composed of various flows, and “flow space” becomes a new perspective for the study of urban and regional structures [5]. As soon as the concept of “flow space” was put forward it received widespread attention, which caused the study of regional spatial structure to change from urban form grade into that of urban network structure, function and connection [15]. At first, scholars used postal data, freight traffic data, population flow data and other data to study urban network structure and network hierarchy [16,17]. Mitchelson et al. took US postal data as the information flow and judged the economic connections between American cities from the perspective of information flow [18]. Based on flow characteristics of the high-end service industry, Peter Taylor et al. set up the urban network framework with global cities as the research scope [15]. Derudder, B. et al. took air passenger flow information as the data to evaluate the world network spatial pattern [19]. They pointed out the defects of previous assessments and constructed a global intercity matrix to avoid the shortcomings of previous studies. Cai et al. took daily long-distance passenger arrivals and departures as traffic passenger flow data to study the regional function polycentric characteristics of the Pearl River Delta urban agglomeration [20]. Ma et al. analyzed the regional spatial pattern of the Yangtze River Delta based on population flow data [21]. 

Furthermore, the information flow represented by Internet big data has become a new direction of regional integration research from the perspective of “flow space” [22,23]. Xiong et al. obtained user-attention data among cities in the Yangtze River Delta based on the Baidu index and analyzed the spatiotemporal evolution of urban networks in the Yangtze River Delta [24]. Based on the data from Sina Weibo, Zhen et al. studied the characteristics of urban network development in China from the perspective of network social space [25]. In order to overcome the problem of unitary selection of flow factors, Qiu et al. conducted a comparative analysis of traffic flow and information flow to explore the spatial network pattern of the Guangdong–Hong Kong–Macao Greater Bay Area [8]. Wang et al. selected human flow, logistics, capital flow and information flow to conduct quantitative discrimination on the regional spatial structure of the Pearl River Delta [26]. These studies combine traditional data flow with information flow and further promote the application and practice of flow space theory. 

In the meantime, the factors driving the development of urban spatial pattern on this basis has been hotly discussed. Urban function and orientation is taken as one of the most important factors in urban development. Samuel points out that explosive urban and political change is driven not only by the newly rich but also by state-driven urban planning processes [27]. Federico uses Poblenou as an example to analyze the important role of government urban planning in urban development or revitalization [28]. What is more, it is said that the occurrence of urban events also affects the development of cities from different aspects. After studying the development layout of Tianjin International Convention and Exhibition Center, Xie et al. found that exhibition economy is a huge engine driving urban development [29]. Zhang et al. took Nanjing Olympic Sports New City as an example to explain the positive role of urban events in enhancing city visibility, driving economic development and improving infrastructure [30]. In addition, historical and cultural factors, as well as the use of these factors, will also affect the change of urban spatial pattern. Federico advocated that historical and cultural sites should be properly developed and utilized in order to achieve cultural, economic, environmental and social sustainability [31]. Los Angeles also thinks about how a historic city should adapt to its historical development [32]. He believes that a city should build its brand on the basis of its history and culture. 

Based on the previous research, this study uses urban network analysis from the perspective of flow space to explore the urban spatial connection and network structure of urban agglomeration in the Yangtze River Delta, which not only enriches the empirical and methodological research of flow space with the application of social network analysis, but also contributes to the study of urban agglomeration and regional networks, providing a scientific basis for regional high-quality development. 

## 3. Study Area, Data Source and Research Method

### 3.1. Study Area

**Figure 1:**
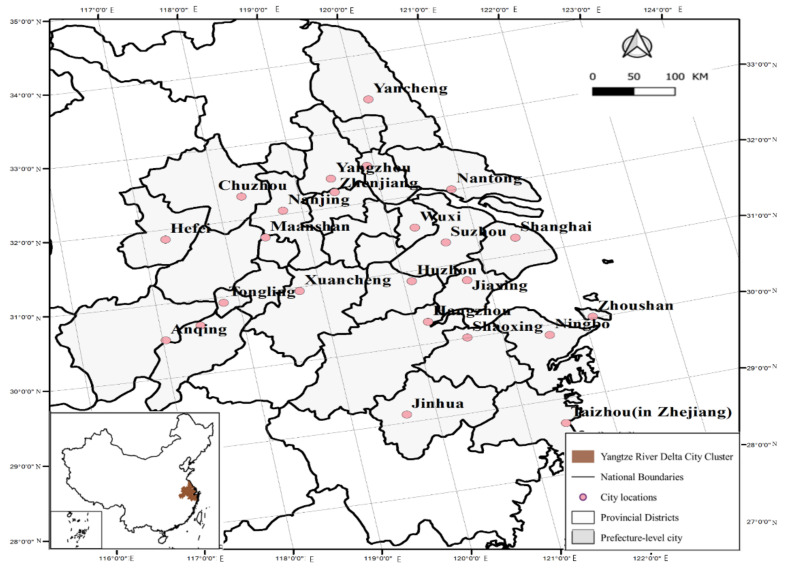
Map of urban agglomeration in the Yangtze River Delta.

The Yangtze River Delta Urban Agglomeration, with Shanghai as the center, is located in the alluvial plain in eastern China before the Yangtze River enters the sea. It is an important intersection zone of “One Belt And One Road” and the Yangtze River Economic Belt. It plays a pivotal strategic position in the macroeconomic development pattern of China [33]. The research scope of this paper is the urban agglomeration in the Yangtze River Delta as determined in *The Planning for the Development of the Yangtze River Delta Urban Agglomeration*, and the research unit is the prefecture-level administrative region. The specific area scope is shown in Figure 1.

### 3.2. Data Sources

The Baidu index measures the number of searches that residents of one city make regarding their interest in another city. According to the statistical report released by StatCounter traffic website in 2018, the utilization rate of Baidu search engine in China is 70.3%, accounting for first place. It can truly reflect the residents’ attention to another city in the region. Based on this, this paper selects the Baidu search index of each city in the Yangtze River Delta urban agglomeration region as the information-flow data and obtains the 2015 and 2019 Baidu search index matrix of the Yangtze River Delta urban agglomeration.

### 3.3. Research Method

According to the research ideas of this paper, this part first introduces how to calculate the intensity of information flow and the calculation method for difference coefficient. Secondly, social network analysis methods are introduced, including network density analysis, network centrality analysis and condensed subgroup analysis. Finally, the multiple regression model used in this paper is introduced. 

#### 3.3.1. Information-Flow-Strength Calculation Formula

(1) Information-flow-strength calculation formula

The calculation formula for information-flow intensity refers to the research method of information-flow intensity used by scholar Peter Taylor, in the related research of world urban related networks [15]. The intensity of information flow between two cities is represented by the product of network attention. The calculation formula is as follows:

(1) $$R_{ij}=U_{ij}\times U_{ji}$$

(2) $$R_{i}=\sum_{j=1}R_{ij}$$

where $R_{ij}$ is the information flow between city *i* and city *j*. $U_{ij}$ represents the information attention of city *i* to city *j*. $\;U_{ji}$ represents the information attention of city *j* to city *i*. $R_{i}$ represents the total information flow of city *i*.

(2) Diversity factor

The coefficient of variation is the percentage of the standard deviation of a group of data and its mean value. It is a relative indicator for measuring the degree of dispersion of data and a relative difference quantity. Because the relative difference quantity does not have a unit of measurement, it is suitable for the comparison of data variation with different units of measurement, or the same units of measurement but large differences in concentrated quantities. In this paper, the difference coefficient is used to calculate and compare the degree of difference in a series of Baidu indices in different cities in different time periods. The larger the value is, the greater the degree of difference within the series is and the higher the degree of data dispersion is. The calculation formula is as follows:

(3) $$C_{v}=\frac{\delta }{x}=\frac{\sqrt{\frac{\sum_{i=1}^{n}{\left(x_{i}-x\right)}^{2}}{n-1}}}{x}$$

where $\;C_{v}\;$ is the difference coefficient.  δ  is the standard deviation of the sequence.  x is the average of the sequence.  n  is the number of the sequence, and $\;x_{i}\;$ is the value of the *i*th number of the sequence.

#### 3.3.2. Social Network Analysis Methods

Social Network Analysis (SNA) is a research branch gradually developed in sociology, psychology, anthropology, mathematics, communication science and other fields since the 1970s, which is used to study the associations between multiple actors [34]. In social network analysis, the “network” is considered to be a series of social relations connecting actors. It adopts the method of quantitative analysis to model the connections between people and estimate the parameters, so that the network structure becomes intuitive [35]. In this paper, taking cities as nodes, the relationship network of urban agglomeration in Yangtze River Delta is constructed, and the social network structure is analyzed from three aspects: network density, network centrality and cohesive subgroup.

(1) Network density

Network density is an index used to describe the interconnection density of nodes in a network. The network density value specifically represents the ratio of the number of connections established by each node to the maximum number of potential connections. The larger the network density value is, the closer each node in the network is, and the greater the influence of the whole network on each node in the network is. Its calculation formula is as follows:

(4) $$D=\sum_{i=1}^{i=k}\sum_{j=1}^{j=k}d\left(n_{i},n_{j}\right)/k\left(k-1\right)$$

where *D* is the network density. *K* is the number of node cities. $d\left(n_{i},\;n_{j}\right)$ is the relationship quantity established between $n_{i}$ and $n_{j}$. If there is a direct connection relationship between city *i* and city *j*, $d\left(n_{i},\;n_{j}\right)$ is assigned as 1. Otherwise, it is assigned as 0. For the urban agglomeration network, the greater the network density value, the more frequent the communication between cities in the urban agglomeration.

(2) Network centrality

Centrality is an index used to explore the centralization level of the overall network. Existence centrality, intermediary centrality and proximity centrality can reflect the position of urban nodes in the network. Degree centrality can directly measure the importance of nodes in the network. The higher the degree centrality of a node is, the higher its level is in the overall network. The calculation formula is as follows:

(5) $$C_{D}\left(C_{i}\right)=d\left(C_{i}\right)/\left(n-1\right)$$

where $C_{D}(C_{i}$) is the centrality degree of a node. *d*($C_{i}$) is the node degree, and *n* − 1 represents the total number of lines starting from a node.

(3) Cohesive subgroups

When certain actors in a network are so closely related that they combine into a sub-group, such a group is called a cohesive subgroup in social network analysis. The condensed subgroup analysis aims to analyze how many such subgroups exist in the network, the characteristics of the relations among the members of the subgroups and the characteristics of the relations between the members of one subgroup and the members of another subgroup. This analysis seeks to explore how the overall structure of the network is composed of small group structures, and to judge the condensed subgroups in the overall network according to the categories of “relatively strong, direct, close, frequent or positive relationships” among the nodes in the subgroups. Its form can be measured from the reciprocity of the relationship, the proximity or accessibility among the members of the subgroup, the frequency of the relationship among the members of the subgroup, and the density of the relationship among the members of the subgroup.

#### 3.3.3. Multiple Linear Regression Model

Exploring the influence of different factors on the urban network structure is helpful to identify the restrictive factors of urban development and to find the path to improve the urban network. By analyzing the influencing factors of the urban network structure through multiple linear regression, this paper has more practical significance.

The model constructed in this paper is as follows:

(6) $$y=\beta_{0}+\beta_{1}x_{1}+\beta_{2}x_{2}+\ldots \ldots +\beta_{n}x_{n}+\varepsilon$$

where y represents the explained variable. x  represents the explanatory variable. n  represents the number of explanatory variables. ε  represents the random disturbance term. β represents the unknown parameter in the model, and the level of β value represents the influence of the explanatory variable on the dependent variable.

## 4. Results and Analysis

### 4.1. Spatiotemporal Variation of Information-Flow Connection

#### 4.1.1. The Total Amount of Information Flow and Its Evolution

**Figure 2:**
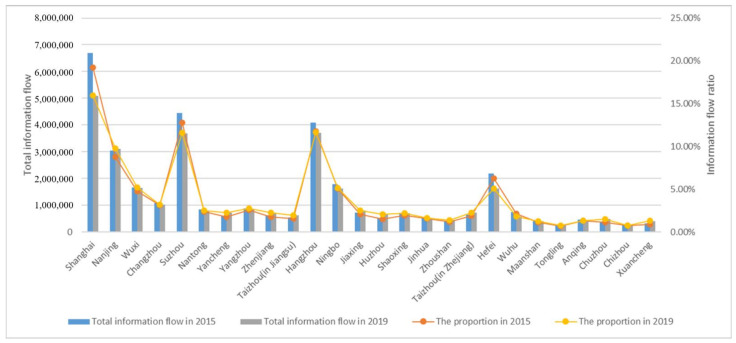
Total information flow and its proportion.

(1) According to the calculation of the total information flow, the total information flow of the cities in the Yangtze River Delta urban agglomeration in 2015 was 348, 172, 02, and the total information flow of the cities in the Yangtze River Delta urban agglomeration in 2019 was 318, 140, 78. The total information flow of the Yangtze River Delta urban agglomeration showed a downward trend. The trend is different from previous studies. The reason is that during 2015–2016, a major event in some cities in the Yangtze River Delta urban agglomeration led to abnormally high total flow values, such as the major stampede in Shanghai in 31 December 2014. The search volume rose sharply in January 2015. Similarly, search volume increased due to other events. As a result, the total information flow in 2015 is higher than that in 2019.
(2) As can be seen from Figure 2, the total amount of information flow of Shanghai, Suzhou and Hangzhou is much higher than that of other small and medium-sized cities in the Yangtze River Delta urban agglomeration. It can be seen that Shanghai, Suzhou and Hangzhou are the core cities in the Yangtze River Delta urban agglomeration, and they have the most frequent information flow with other cities. From the perspective of interannual change, the proportion of the total information flow of the three cities in the total information flow of the 26 cities in the Yangtze River Delta urban agglomeration decreased from 43.7% in 2015 to 39.2% in 2019. With the development of surrounding secondary cities, Shanghai, Suzhou and Hangzhou still maintain the status of core cities. However, from the proportion of information flow, we can see that the scope of the core-city circle in the Yangtze River Delta extends outward.
(3) In 2015, the variation coefficient of the total information flow in the Yangtze River Delta urban agglomeration was 0.868, which was less than 1, indicating that the dispersion degree in the Yangtze River Delta urban agglomeration was low and the variation degree in the data was small. However, in 2019, the coefficient of variation was 1.006, slightly greater than 1, indicating that the degree of dispersion within the Yangtze River Delta urban agglomeration has expanded over time, and the gap of information correlation among cities shows an expanding trend. The combination of (2) shows that the core urban circle of the Yangtze River Delta urban agglomeration has expanded from 2015 to 2019, but the information-flow gap between the peripheral cities, such as Tongling and Xuancheng, and the central cities and subcentral cities has gradually widened, and there is still a certain polarization phenomenon in the Yangtze River Delta urban agglomeration.

#### 4.1.2. Characteristics of Network Hierarchy

**Figure 3:**
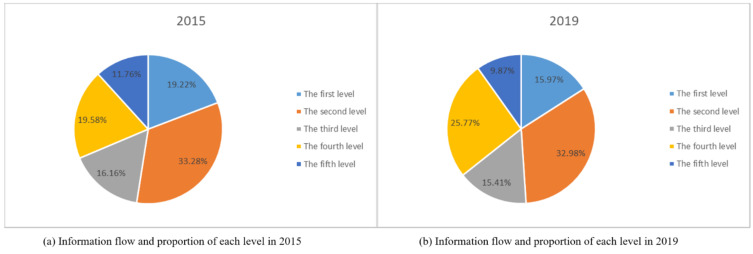
Information flow and proportion of each level: (**a**) 2015; (**b**) 2019.

**Figure 4:**
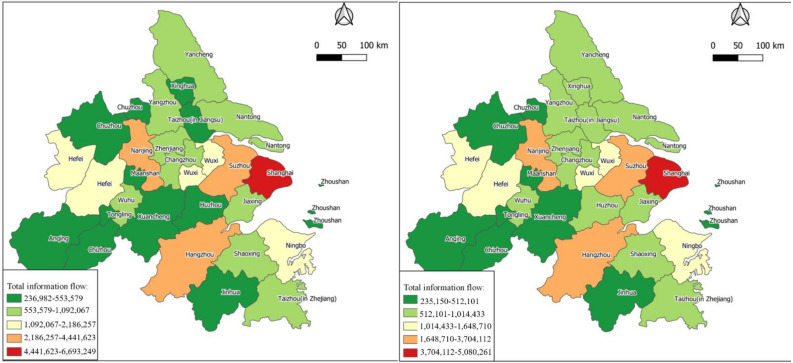
Level of total information flow.

In order to explore the spatiotemporal variation characteristics of the urban network in the Yangtze River Delta urban agglomeration, the network hierarchy of cities in the Yangtze River Delta urban agglomeration was divided by the natural breakpoint method of ArcGIS software (Environmental Systems Research Institute, RedLands, CA, USA) based on total information-flow data in 2015 and 2019. As shown in Table 1, The information flow and proportion of each level is shown in Figure 3. the urban network levels of the Yangtze River Delta urban agglomeration in 2015 and 2019 are visualized, as shown in Figure 4.

As can be seen from the above chart, the network hierarchy distribution characteristics of the Yangtze River Delta urban agglomeration are as follows:

(1) The overall structure of the regional network level of the Yangtze River Delta urban agglomeration is stable. In 2015 and 2019, the cities in the first three levels of the Yangtze River Delta urban agglomeration are exactly the same. In 2015, there were nine cities in the fourth level, while in 2019, Huzhou and Taizhou were added to the fourth level. By comparing the ratio of the total amount of information flow at each level to the total amount in Figure 3 in 2015 and 2019, it can be found that the ratio of each level changes numerically, but the change range is very small. It shows the overall coordinated development of the Yangtze River Delta urban agglomeration region, where only a few cities display high-speed development across the hierarchy.
(2) The overall hierarchical distribution of regional cities presents a pyramid shape, the higher the hierarchy, the fewer the number of cities. As the top of the pyramid of regional city level, Shanghai, together with Hangzhou, Suzhou and Nanjing in the second level, forms a model of “one superpower and three cores”. The total information flow of these four cities accounted for 52.5% and 48.9% of the total information flow of the Yangtze River Delta region in 2015 and 2019, respectively. It shows that these four prefectures have established close links with other prefectures in the region, and the total amount of information flow has an absolute leading position compared with other cities in the region, occupying an absolute core position in the urban agglomeration.

### 4.2. Analysis on the Information-Flow Network Structure

**Figure 5:**
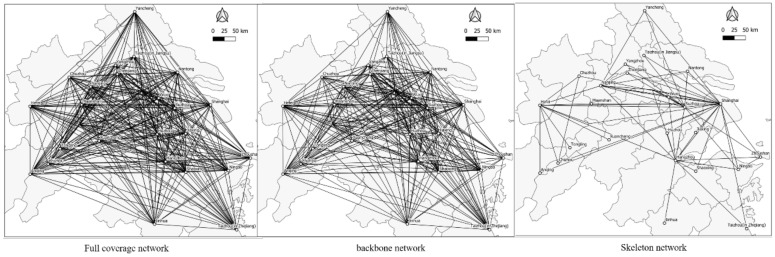
Information-flow network in 2015.

**Figure 6:**
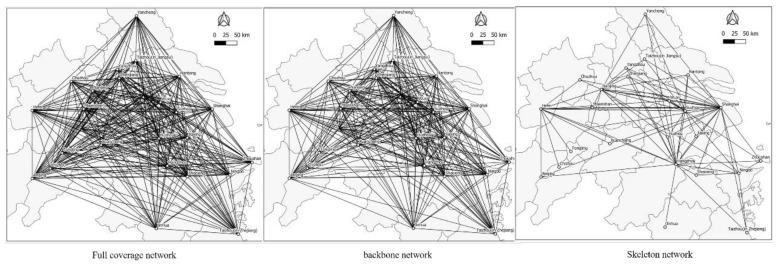
Information-flow network in 2019.

The social network analysis (SNA) is used to further explore the network structure of the Yangtze River Delta urban agglomeration from the perspective of information connection. The strength matrix of information-flow connection between the cities of the Yangtze River Delta urban agglomeration in 2015 and 2019 was used to draw and form the network structure diagram as shown in Figure 5 and Figure 6.

In the network structure shown in Figure 5 and Figure 6, the endpoint represents the node cities within the region. When the node connection between the two cities is greater than the set threshold, a line will be generated between the nodes. In order to observe the network structure hierarchy within the region more clearly, the full coverage network of the Yangtze River Delta urban agglomeration is constructed according to the standard that the node connection of information flow between two cities is greater than 0. The backbone network of urban agglomeration is constructed according to the standard that the connection of information-flow nodes between two cities is greater than 100. The skeleton network of urban agglomeration is constructed according to the standard that the information-flow node connection between the two cities is greater than 300.

By comparing Figure 5 and Figure 6, it can be concluded that from the perspective of the skeleton network, the network structure of the Yangtze River Delta urban agglomeration in 2015 and 2019 is basically a “triangle” structure with Shanghai, Hangzhou, Hefei, Nanjing and other provincial capitals as the most critical nodes, and Nanjing is located on one of the bottom lines of the “triangle” network. The information-flow network connection in 2019 is significantly higher than that in 2015, indicating that the information network connection among urban agglomerations in the Yangtze River Delta has become closer with time.

#### 4.2.1. Network Density Analysis

**Figure 7:**
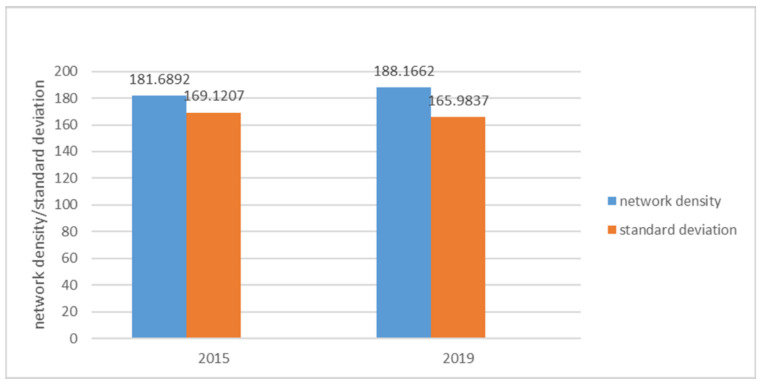
Network density value.

The network density between urban agglomerations can reflect the degree of close connection between cities. In this paper, the density analysis software tool UCINET was used to calculate the information-flow network density of the Yangtze River Delta urban agglomerations in 2015 and 2019. The calculation results are shown in Figure 7. In 2015, the network density and standard deviation of information flow in the Yangtze River Delta urban agglomeration were 181.6892 and 169.1207, respectively. The network density of information flow in 2019 was 188.1662, and the standard deviation is 165.9837. The network density among urban agglomerations is relatively high, indicating close information connection among members, and the connection density in 2019 was higher than that in 2015. At the same time, the standard deviation of information-flow network density decreased with time, indicating that the gap of information-flow connection strength between regional inland cities decreased. The relationship between cities in the Yangtze River Delta is closer and the gap between cities is gradually decreasing.

#### 4.2.2. Network Centrality Analysis

**Figure 8:**
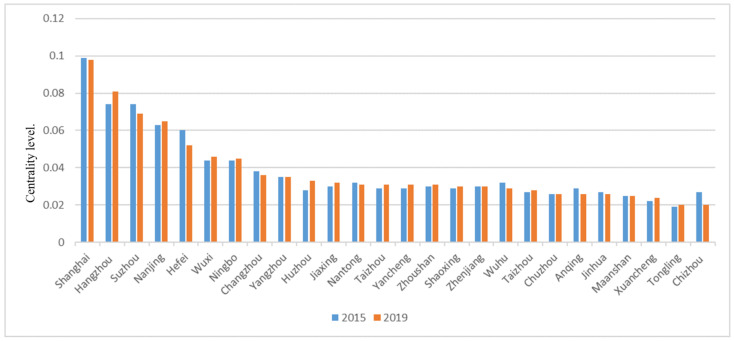
Centrality level.

Figure 8 shows the degree of centrality histogram constructed using the information-flow connection matrices of the Yangtze River Delta urban agglomeration in 2015 and 2019.

Figure 8 shows that from the ranking of centrality values, Shanghai, Hangzhou, Suzhou, Nanjing and Hefei are significantly higher than other cities in the two years, indicating that these five cities occupy a central position in the Yangtze River Delta urban agglomeration network, have development advantages, and have a greater role in promoting the surrounding cities. From the perspective of interannual change, the value of city center degree has little change in general, and there is no significant change in the city center degree. It can be seen that the development center of urban agglomeration in the Yangtze River Delta is still concentrated in large- and medium-sized cities. Although the center degree of Shanghai, Suzhou and other cities has decreased, it is still significantly higher than that of other cities.

#### 4.2.3. Analysis of Condensed Subgroups

**Figure 9:**
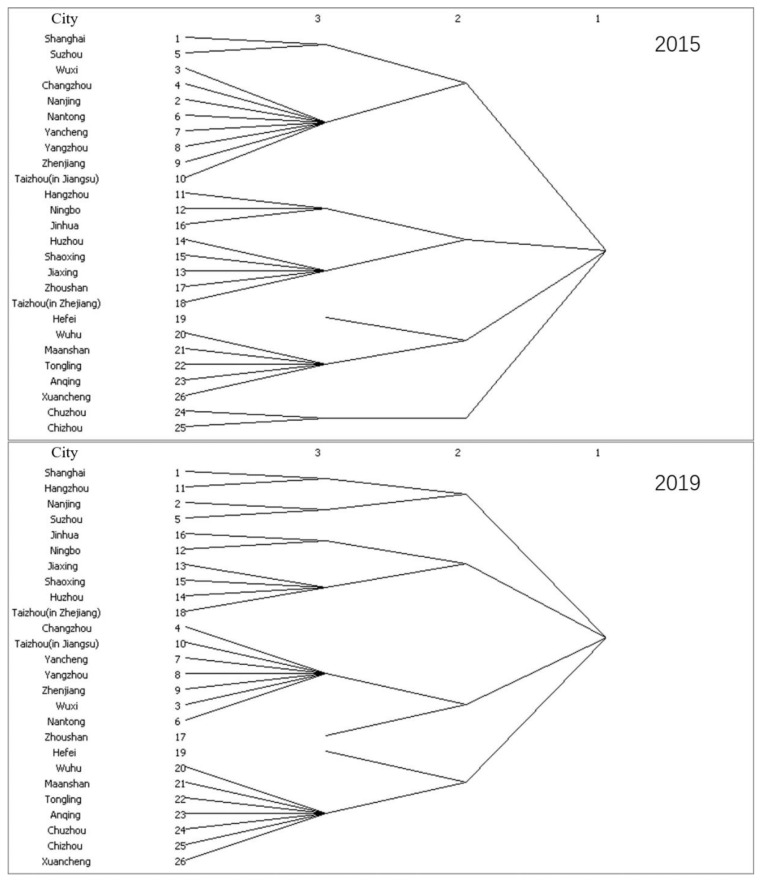
Cohesive subgroups.

**Figure 10:**
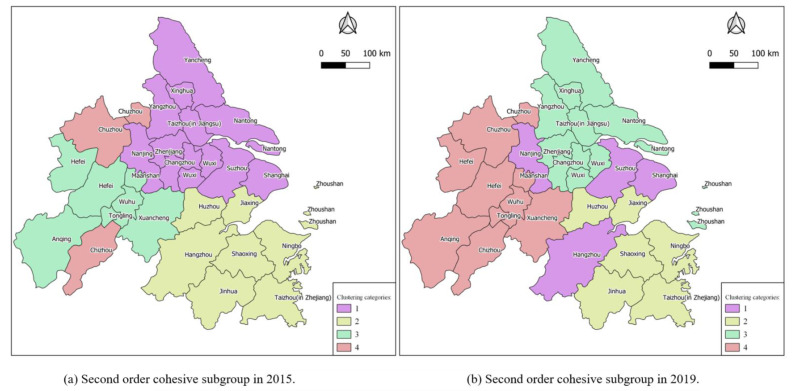
Second order cohesive subgroup: (**a**) 2015; (**b**) 2019.

**Figure 11:**
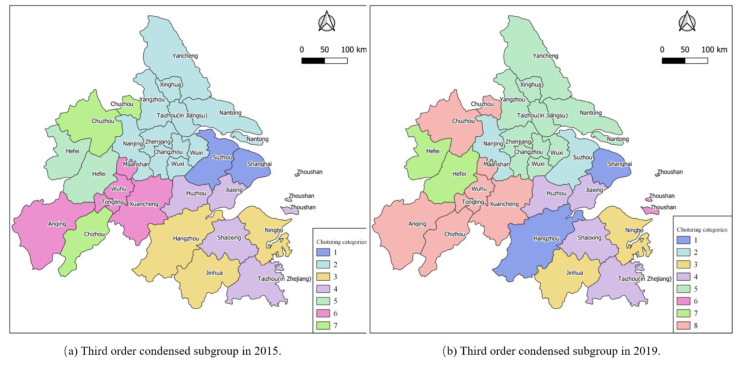
Third order condensed subgroup: (**a**) 2015; (**b**) 2019.

The agglomerated subgroup analysis divides the node cities with relatively strong connections into a subset, which can explore the internal structure of the urban network from a more microscopic perspective. In this paper, the structure diagrams of agglomerated subgroups of Yangtze River Delta city groups in 2015 and 2019, as shown in Figure 9, are obtained, and the agglomerated subgroups of level two and level three are visualized as shown in Figure 10 and Figure 11.

It can be seen from Figure 9 that subgroups in the Yangtze River Delta urban agglomeration are obviously agglomerated. In 2015, the information-flow network in the region formed four large two-level subgroups and seven small three-level subgroups. As shown in Figure 10 and Figure 11, agglomeration of urban small groups is obvious in the urban agglomeration, and the agglomeration subgroups formed at the secondary level are adjacent to each other, indicating that the geographical location has a significant restriction effect on the information-flow network. Further subdivision shows that the agglomerated subgroups formed at the three-level level are spatially separated, indicating that the development of information and network makes the information flow break through the geographical distance to some extent.

### 4.3. Analysis of the Influencing Factors of Information-Flow Network

#### 4.3.1. Variable Selection and Model Construction

(1) Variable selection

In this paper, the total amount of information flow in 2019 is selected as the explained variable. The selection of the explanatory variable takes into account the quantification, accessibility and correlation of the data, draws on the existing studies of some scholars, and comprehensively considers the possible influencing factors of the connection strength of information flow in the Yangtze River Delta urban agglomeration. This paper selects explanatory variables from three aspects: economic scale, population scale and infrastructure.

Specific indicators of explanatory variables are as follows: gross regional product (GDP), resident population (population) and Number of Internet broadband subscribers (inter). Explanatory variable data in this paper were obtained from the *China Urban Statistical Yearbook 2020*. Specific descriptions of relevant variables and descriptive statistics are shown in Table 2 and Table 3.

(2) Model building

This model first assumes that there is a multivariate linear correlation between explanatory variables and explained variables, and tests whether the hypothesis is valid in the process of regression analysis. The specific regression model is as follows:

(7) $$y=\beta_{0}+\beta_{1}x_{1}+\beta_{2}x_{2}+\beta_{3}x_{3}+\varepsilon$$

where *y* is the explained variable. That is, it represents the older River Delta urban agglomeration flow around the city in 2019. $\beta_{1}$ is constant. $x_{1}\;$ represents regional GDP. $x_{2}\;$ is on behalf of the resident population. $x_{3}$ represents the number of broadband users in the region. $\beta_{n}$ represents the degree of influence of each explanatory variable on the total information flow of the explained variable, and ε is the random error term.

#### 4.3.2. Analysis of Regression

(1) Correlation analysis between the total amount of information flow and each influencing factor

Through SPSS (International Business Machines Corporation, Armonk, NY, USA), bilateral correlation analysis was conducted for the data of each indicator, and the results were shown in Table 4.

Observing the results in Table 4, it can be seen from the Pearson correlation coefficient that the gross regional product, the permanent resident population of a region and the number of Internet broadband access users are all positively correlated with the total amount of urban information contact, and the gross regional product and the number of broadband access users have a strong correlation and a strong influence. As can be seen from the significance level, the three indicators belong to the significant data indicators. Therefore, the three indicators were used as multiple linear regression to explore the influence of each variable on the explanatory variable under the simultaneous action of multiple variables.

(2) Multiple regression analysis

The regression results were shown in Table 5, the model goodness of fit $R^{2}$ is 0.931, which shows that the model fitting result is good. The value of F statistic is 99.75, greater than the critical value. The overall model passed the significance test. Specifically, GDP index passed the test of significance at the 1% level. The area resident population index is at the 20% significance level by significance test. Internet broadband access subscribers index is at the 20% significant level by significance test. These three indicators have the most obvious influences on the flow of information. Therefore, the regression equation is as follows:


(8) y =−34285.25+178.64gdp −663.254popul +1450.541inter + ε

The regression coefficient of GDP is 178.64. That is, for every CNY 100 million increase in GDP, the total amount of information flow increases by 178.64. The regression coefficient of the number of Internet broadband access users is 1450.541. That is, every increase in the number of Internet broadband access users by 10,000 households, the total information flow increases by 1450.541. The coefficients of these two explanatory variables are both positive, indicating that the increase in these two explanatory variables in a certain area will increase the total amount of information flow in the region. In particular, the regression coefficient of the resident population in the region is −663.254, which is negative. The increase in the resident population in the region tends to reduce the total information flow.

### 4.3.3. Analysis of Other Influencing Factors

The factors influencing the total amount of regional information flow were investigated from the three aspects of regional gross domestic product, permanent resident population and Internet broadband user access. However, the regional information-flow network is also affected by other qualitative factors. Therefore, this section will briefly analyze other factors influencing the urban network.

(1) Urban function and its orientation

In urban agglomeration, different cities play different roles and occupy different positions. Different functions and positioning of cities will affect the degree of concern of cities. In the Yangtze River Delta urban agglomeration, the total information flow of Shanghai takes first place. As a municipality directly under the central government of China, Shanghai plays an important leading role in politics, economy, culture and other aspects. It has a significant functional status as a core city and occupies a core position in the Yangtze River Delta urban agglomeration.

Nanjing, Hangzhou and Hefei are the administrative and economic centers of the three provinces, respectively. Suzhou, which is adjacent to Shanghai, is also the manufacturing center of the Yangtze River Delta and plays a pivotal role in the region. In the Yangtze River Delta region, the amount of information searching of the above four cities is much higher than that of other cities in the same region. It can be seen that the higher the function and status of a city, the more attention it receives. Therefore, it has a higher amount of information searching, and has more potential to become the core node of urban agglomeration.

On the contrary, Tongling, Chizhou, Xuancheng and other cities are prefecture-level cities in Anhui Province, not political, economic, or cultural centers of Anhui Province. They have a relatively low status and can only serve a limited range. Therefore, they have attracted limited attention and are less connected than other cities in the information-flow network.

(2) Urban event effect

Events that have a significant impact on a city in a certain period of time are called urban events, such as major sports events, important national and international conferences, etc. Due to their social universality, they will have a substantial impact on the urban material environment, and even bring some changes to the urban spatial structure [29]. For example, in August 2019, the World Artificial Intelligence Conference was held in Shanghai. As a conference with world-class influence, it attracted the attention of the whole country and even the whole world. In November 2019, the second China International Import Expo was held in Shanghai National Convention and Exhibition Center. The expo attracted the attention of people at home and abroad, which greatly improved the city image and attention of Shanghai.

In addition to the major events and conferences listed above, major news events will also arouse public attention and discussion. For example, on New Year’s Eve in 2015, a major stampede occurred at Chen Yi Square on the Bund in Shanghai, resulting in 36 deaths and 49 injuries. This major public security incident has attracted attention from all over Shanghai, and the total amount of information flow in Shanghai increased sharply for several consecutive days.

(3) Historical and cultural factors

The influence of historical and cultural factors on urban development cannot be ignored. The three provinces and one city of Suzhou, Shanghai, Zhejiang and Anhui have adjacent geographical location and similar location conditions. In historical documents, there are many records such as “Wu and Yue are neighbors, the same customs and soil” and “Wu and Yue two states, the same gas and common customs”. From the Eastern Jin Dynasty to the present, we can see that the Yangtze River Delta region is closely connected and has a high basis of historical identity. Since the reform and opening up, the frequent flow of talents and close economic exchanges among those three provinces have laid a solid foundation for the integration of the Yangtze River Delta. Today, the cities of the Yangtze River Delta are integrated with each other in infrastructure, economy, system and mechanism, providing more conditions and opportunities for the high-quality integrated development of the region.

Not only should the influence of historical and cultural factors be considered, how to make use of these historical and cultural factors to promote the positive development of the city is also of great significance. A city should build its brand based on its history and culture [32]. For the Yangtze River Delta region, the existing historical and cultural similarities should be used to better promote the coordinated development of urban agglomeration. 

## 5. Conclusions

Firstly, unlike previous research conclusions, it has been found that Yangtze River Delta urban agglomerations in 2019 had lower total flow than 2015, determined by calculating the total information flow across the Yangtze River Delta urban agglomeration from city to city. This phenomenon is due to the high amount of information flow in 2015 caused by major events. Two years of research data show that the top three cities in the total information flow of the Yangtze River Delta urban agglomeration are Shanghai, Suzhou and Hangzhou. These three cities are the core cities, but the proportion of the total information flow of the three cities has decreased, indicating that the core-city circle of the Yangtze River Delta has expanded to the periphery. Through calculating the coefficient of variation of the Yangtze River Delta urban agglomerations, it was found that the Yangtze River Delta core-city circle range has widened. However, polarization phenomena still exist in the Yangtze River Delta urban agglomeration due to geographical location and economic level.

Secondly, this paper divides the network hierarchy through the natural fracture method and obtains the characteristics of information-flow network hierarchy in the Yangtze River Delta urban agglomeration. The hierarchical structure of information flow network in the Yangtze River Delta urban agglomeration has changed from the distribution pattern of “1 + 3 + 3 + 9 + 10” in 2015 to the distribution pattern of “1 + 3 + 3 + 11 + 8” in 2019, and the number of cities at the fourth level has increased. The information-flow network hierarchy of the Yangtze River Delta urban agglomeration presents a stable “pyramid” pattern, with fewer cities at the top level and more at the bottom level. Over time, the number of cities at the bottom level shows a decreasing trend and develops to a “spindle” type distribution. By comparing the ranking of different cities, it can be found that there are slight changes in the ranking of information flow in different cities.

Thirdly, this paper uses information-flow data to further explore the network structure of the Yangtze River Delta urban agglomeration through social network analysis. The backbone network of the Yangtze River Delta urban agglomeration is a “triangle” structure, with Shanghai, Hangzhou, Hefei and Nanjing as its vertices, and Nanjing is located on one of the bottom lines of the triangle network. The overall network density of the Yangtze River Delta urban agglomeration shows an increasing trend, and the cities within the urban agglomeration exchange more frequently. The network centrality of big cities is relatively significant, with Shanghai, Hangzhou, Suzhou, Nanjing and Hefei ranking top in 2015 and 2019. The phenomenon of agglomeration of urban small groups is obvious in urban agglomeration, and the agglomeration subgroups formed at the second level are adjacent to each other, which indicates that geospatial location has an obvious effect on the information-flow network. The agglomerated subgroups formed at the three-level level are spatially separated, which indicates that the development of information and networks causes the information flow to break through the geographical distance to some extent.

Fourthly, this paper takes the total amount of regional information flow as the explained variable, and selects explanatory variables from the three aspects of economic scale, population scale and infrastructure, to conduct regression analysis on the explained variables. The specific explanatory variables are GDP, resident population and broadband user access. The regression results show that the three aspects have a significant impact on the total information flow of the region, among which the number of Internet broadband users has the largest impact on the total information flow. In addition, urban functions and their positioning, urban events, history and culture, and other factors that are difficult to quantify also have a certain impact on the information-flow network between cities.

### Policy Suggestion

Based on the above research on the information-flow network structure and influencing factors of the Yangtze River Delta urban agglomeration, the following policy suggestions are proposed to promote the optimization of the network structure of the Yangtze River Delta urban agglomeration in view of the network structure characteristics and existing problems.

(1) The radiation effect of central- and sub-central cities on marginal cities should be strengthened. The stronger the comprehensive strength of a city, the larger the network connecting beam established with other cities in the region, and the stronger the radiating and driving role of a city to other cities [36]. The central cities in the Yangtze River Delta urban agglomeration have played a leading role to the subcentral cities, but the radiation effect of the inner marginal cities in the Yangtze River Delta urban agglomeration is less, and there is a trend of polarization within the urban agglomeration. Therefore, the network construction of marginal cities should be further strengthened, and the radiating and driving role of superior cities should be brought into full play.
(2) Optimize the distribution of urban levels within the region. The urban spatial hierarchy of the Yangtze River Delta urban agglomeration presents a relatively stable “pyramid”-shaped distribution and has a tendency to develop into “spindle”-shaped distribution. Cities at the bottom of the pyramid should take the initiative to establish extensive connections with cities at other levels. It is of great importance to seize development opportunities brought about by the information age, improve the attention and influence of cities, move towards a higher city level, and promote the network level of urban agglomeration in the Yangtze River Delta to be a more stable “spindle”-shaped distribution.
(3) Gradually reduce administrative barriers between regions. Under the influence of network and informatization, the information flow between regions has the tendency to break the barrier of administrative division [37]. The results of the analysis of agglomerated subgroups in the Yangtze River Delta urban agglomeration show that the agglomerated subgroups formed in the region are still largely limited by geographical and spatial location. Therefore, the relevant departments of the Yangtze River Delta urban agglomeration should further guide the relevant cities in the region to strengthen exchanges and cooperation in economic, political and cultural aspects, establish a unified market, and break through regional and administrative restrictions.
(4) Improve urban network infrastructure. The number of urban broadband users is the most influential factor affecting the total amount of urban information flow. The level of urban Internet infrastructure is the basic material guarantee for the intensity of urban information flow. Therefore, the government departments and enterprises of each city should respond to the national “Internet +” initiative, improve the construction of regional information network system, and give play to the leading role of information in urban brand and visibility.

## Figures and Tables

**Table 1:** Network hierarchy distribution.

Level	2015	2019 (Changes in Rankings)
The first level	Shanghai	Shanghai
The second level	Suzhou, Hangzhou, Nanjing	Hangzhou (+1), Suzhou (−1), Nanjing
The third level	Hefei, Ningbo, Wuxi	Wuxi (+2), Ningbo, Hefei (−2)
The fourth level	Changzhou, Yangzhou, Nantong, Wuhu, Jiaxing, Shaoxing, Taizhou (in Zhejiang), Zhenjiang, Yancheng	Changzhou, Yangzhou, Jiaxing (+2), Nantong (−1), Yancheng (+4), Zhenjiang (+2), Taizhou (in Zhejiang), Shaoxing (−2), Huzhou (+3), Taizhou (in Jiangsu) (+1), Wuhu (−7)
The fifth level	Jinhua, Taizhou (in Jiangsu), Huzhou, Anqing, Zhoushan, Maanshan, Chuzhou, Xuancheng, Chizhou, Tongling	Jinhua (−2), Chuzhou (+3), Zhoushan, Anqing (−2), Xuancheng (+1), Maanshan (−2), Tongling (+1), Chizhou (−1)

**Table 2:** Variable declaration.

	Indicators	Units
Explained variable	y	
Explaining variable	gdp	billion
	population	ten thousand people
	inter	ten thousand family

‘y’ is the total amount of information flow.

**Table 3:** Descriptive statistics.

Indicators	Units	Mean	Max.	Min.	Standard Deviation
y		1,223,618	5,080,261	235,150	1,230,950
GDP	billon	7213.18	32,679.87	831.73	6971.51
Population	ten thousand people	567.46	2428.14	96.6	440.74
Inter	ten thousand family	238.35	588	41	157.59

‘y’ is the total amount of information flow.

**Table 4:** Correlation analysis.

	y	GDP	Population	Inter
Pearson Correlation Coefficient	1	0.956 **	0.861 **	0.908 **
Sig.		0.000	0.000	0.000
*N*	26	26	26	26

** indicates that it is significant at the significance level of 5%. ‘y’ is the total amount of information flow. Sig. Represents the statistical *p* value, if *p* value is 0.01 < *p* < 0.05, it is statistically significant; if *p* < 0.01, it is statistically extremely significant.

**Table 5:** Regression result.

Indicators	Result
GDP	178.64 *** (4.24)
Population.	−663.25 (−1.39)
Inter.	1450.54 (1.36)
R square	0.93
Adjusted R square	0.92
F-statistic	99.75

*** indicate that it is significant at the significance level of 1%.

## Data Availability

The data presented in this study are available on request from the corresponding author. The data are not publicly available due to privacy.

## References

[1] Zhuang Y. (2021). Globalization and regionalization: Dual trends and internal logic of contemporary urban development. Soc. Sci. J..

[2] Fang C. (2013). Scientific Basis and Frame System of Urban Development Pattern Optimization in China. Economic Geography..

[3] Guo Q. (2020). Network analysis of northwest Cities from the perspective of flow space. Master’s Thesis.

[4] Chen C., Xiu C. (2014). Research on City Network of Northeast China Based on Space of Flows Areal Research and Development. Areal Res. Dev..

[5] Castells M. (1989). The Informational City: Information Technology, Economic Restructuring, and the Urban-Regional Process.

[6] Hu D. (2002). Castell, Manuel: The Informationized City. Natl. New Bibliogr..

[7] Song J. (2010). Agglomeration Economies of China’s Three Major Urban Agglomerations, 1994–2008. Int. Area Rev..

[8] Qiu J., Liu Y., Chen H. (2019). Urban Network Structure of Guangdong-Hong Kong-Macao Greater Bay Area with the View of Space of Flows: A Comparison between Information Flow and Transportation Flow. Econ. Geogr..

[9] Fan J. (2004). Integration, regional specialization and spatial transfer of manufacturing in Yangtze River Delta. Manag. World.

[10] Chen W., Liu W. (2020). Spatial organization evolution of railway passenger transportation in the perspective of “space of flow”: A case study of the Yangtze River Delta urban agglomeration. Geogr. Res..

[11] Neal Z.P. (2011). From central places to network bases: A transition in the u.s. urban hierarchy, 1900–2000. City Community.

[12] Pan K., Cao Y. (2017). Evolution and Spatial Structure of Container Liner Network in the Yangtze River Delta. Sci. Geogr. Sin..

[13] Wu Q., Ning Y. (2012). Analysis of Urban spatial Network in China: From the perspective of production network of electronic information enterprises. Geogr. Res..

[14] Zhong Y., Wu S., Feng X. (2020). Network Structure Characteristics of Middle Yangtze Urban Agglomeration From the Perspective of Multi-flow. J. Jiangxi Norm. Univ. (Philos. Soc. Sci. Ed.).

[15] Taylor P.J. (2010). Specification of the World City Network. Geogr. Anal..

[16] Xue F., Li M. (2020). Centrality and Symmetry of People Flow Network Structure of the Yangtze River Delta Urban Agglomeration at Multi-Spatial Scales. Econ. Geogr..

[17] Song W., Li X. (2008). Patterns of spatial interaction and hierarchical structure of Chinese cities based on intercity air passenger flows. Geogr. Res..

[18] Mitchelson R.L., Wheeler J.O. (1994). The Flow of Information in a Global Economy: The Role of the American Urban System in 1990. Ann. Assoc. Am. Geogr..

[19] Derudder B., Witlox F. (2005). An Appraisal of the Use of Airline Data in Assessing the World City Network: A Research Note on Data. Urban Stud..

[20] Cai L., Ma G. (2013). Characteristics of Functional Polycentricity of PRD Urban Region Based on Passenger Traffic Flow. Econ. Geogr..

[21] Ma X., Zhang Z. (2020). Study on spatial Evolution characteristics and Situation of Yangtze River Delta Based on Population Flow. Urban Plan. Forum.

[22] Laharotte P.-A., Billot R., Come E., Oukhellou L., Nantes A., El Faouzi N.-E. (2015). Spatiotemporal Analysis of Bluetooth Data: Application to a Large Urban Network. IEEE Trans. Intell. Transp. Syst..

[23] Li S., Peng Z. (2020). Spatial Organization of the Chengyu Urban Agglomeration and its Implications on Planning from the Perspective of Information Flow: City Network Analysis Based on Baidu Index. J. Hum. Settl. West China.

[24] Xiong L., Zhen F. (2013). The Research of the Yangtze River Delta Core Area’s City Network Characteristics Based on Baidu Index. Econ. Geogr..

[25] Zhen F., Wang B., Chen Y. (2012). China’s City Network Characteristics Based on Social Network Space: An Empirical Analysis of Sina Micro-blog. Acta Geogr. Sin..

[26] Wang S., Gao S. (2019). Spatial structure of the urban agglomeration based on space of flows: The study of the Pearl River Delta. Geogr. Res..

[27] Mcelroy E. (2019). Samuel stein 2019: Capital city: Gentrification and the real estate state. London: Verso. Int. J. Urban Reg. Res..

[28] Camerin F. (2019). From “ribera plan” to “diagonal mar”, passing through 1992 “vila olímpica”. How urban renewal took place as urban regeneration in poblenou district (Barcelona). Land Use Policy.

[29] Xie Q. (2018). Research on the Development of Industrial Space Follow the Big City Event: Take Tianjin National Exhibition Center for Example. Anthology of Urban Development and Planning.

[30] Zhang J., Yin J., Luo Z. (2007). Analysis of Urban Growth Manchine Based on Great Event Marketing: Case Study of Nanjing New Olympic City. Econ. Geogr..

[31] Camerin F., Camatti N., Gastaldi F. (2021). Military barracks as cultural heritage in italy: A comparison between before-1900- and 1900-to-1950-built barracks. Sustainability.

[32] (2021). Historic Cities: Issues of Urban Conservation, edited by Jeff Cody and Francesco Siravo. Getty Conservation Institute, Los Angeles, 2019. 610 pp. $60.00. isbn9781606065938. Built Herit..

[33] Tang C., Dou J. (2020). Functional Network Structure and Regional Integration Degree of Yangtze River Delta Urban Agglomerations. Inq. Into Econ. Issues.

[34] Liu J. (2009). Lecture on Whole Network Approach: A Practical Guide to UCINET.

[35] Liu D., Yang Y. (2020). Study on Planning and Policy System of Regional Integration Development in Yangtze River Delta. Environ. Prot..

[36] Li Z., Yao Q. (2019). Economic Globalization, City Network and the Development of Global Cities Journal of East China Normal University. Humanit. Soc. Sci..

[37] Wang Z., Yang S. (2017). Identification of Urban Agglomerations Deformation Structure Based on Urban-flow Space: A Case Study of the Yangtze River Delta Urban Agglomeration. Sci. Geogr. Sin..

